# Using Two Disability Measures to Compare Physical Inactivity Among US Adults With Disabilities

**DOI:** 10.5888/pcd15.170261

**Published:** 2018-01-18

**Authors:** Dana Olzenak McGuire, Kathleen B. Watson, Dianna D. Carroll, Elizabeth A. Courtney-Long, Susan A. Carlson

**Affiliations:** 1 Division of Nutrition, Physical Activity, and Obesity, Centers for Disease Control and Prevention, Atlanta, Georgia; 2 Epidemic Intelligence Service, Centers for Disease Control and Prevention, Atlanta, Georgia; 3 Division of Human Development and Disability, Centers for Disease Control and Prevention, Atlanta, Georgia; 4 Commissioned Corps, US Public Health Service, Atlanta, Georgia

## Abstract

Prevalence of health behaviors among adults with disabilities may vary by disability measure. We used data from the 2011–2015 National Health Interview Survey to estimate prevalence of physical inactivity by disability status using 2 measures of disability: Basic Actions Difficulty questions (BADQ) and a standard 6-question measure (6Q). Disability prevalence (BADQ, 31.1%; 6Q, 17.5%) and inactivity prevalence among adults with disability (BADQ, 42.9%; 6Q, 52.5%) and without disability (BADQ, 24.3%; 6Q, 26.2%) varied by measure; however, both measures highlight inactivity disparities for adults with disability. Disability measures influence physical inactivity estimates and are important for guiding surveillance and health promotion activities for adults with disabilities.

## Objective

Adults with disability represent a substantial proportion of the US population and have a higher prevalence of certain health risks and behaviors, such as physical inactivity, than those without disability (1,2). To monitor the health of people with disability, several questions measuring disability status are used in public health surveys (3,4). Disability prevalence estimates vary on the basis of the measure used (3,5), but little is known about how the prevalence of health-related behaviors, such as physical inactivity, vary by disability measure. We compared the prevalence of inactivity among adults with disability using 2 measures. Understanding how differences in prevalence estimates relate to the measures used to determine them can help better prioritize resources and planning programs to improve the health of adults with disabilities.

## Methods

The National Health Interview Survey (NHIS) is a nationally representative survey of the civilian, noninstitutionalized US population. NHIS collects basic health and demographic information from all family members in a household and additional information from one sample adult. From 2011 through 2015, sample adult response rates ranged from 55% in 2015 to 66% in 2011. NHIS surveys and sampling descriptions are available (www.cdc.gov/nchs/nhis/data-questionnaires-documentation.htm).

NHIS asks sample adults questions related to participation in light-intensity to moderate-intensity or vigorous-intensity leisure time physical activity; inactivity was defined as reporting no leisure time physical activity that lasted at least 10 minutes per week. Disability was defined using 2 measures. The first measure, Basic Actions Difficulty questions (BADQ), uses 17 questions to assess function in 5 domains: movement, hearing, vision, emotion, and cognition. The other measure, 6 standard questions (6Q), was first used in the American Community Survey and incorporated into many national surveys (6). 6Q assesses serious difficulty in at least 1 of 4 functional areas (hearing, vision, cognition, and ambulation [ie, walking or climbing stairs]) and any difficulty in independent living (self-care and community independence). All sample adults were assessed with BADQ, and half of the sample was assessed with 6Q, for which questions were answered by either the person with a disability or his or her designated family member. Individuals identified as having a disability in any BADQ domain (7) or answering yes to any of the 6Q were considered to have a disability.

To account for selection and nonresponse biases, data from the sample of all adults (BADQ) and the half sample of adults (6Q) were weighted so that both data sets would be nationally representative. Prevalence and 95% confidence intervals for disability and inactivity were estimated overall and by demographic characteristic. Pairwise *t* tests and orthogonal polynomial contrasts were used to identify demographic differences and trends in prevalence. SUDAAN release 11.0.0 (RTI International) was used to account for the complex survey design.

## Results

Overall, 31.1% of noninstitutionalized US adults reported a disability by BADQ and 17.5% by 6Q (Table 1). Inactivity prevalence for both measures was higher for individuals with disability (BADQ, 42.9%; 6Q, 52.5%) than individuals without disability (BADQ, 24.3%; 6Q, 26.2%), and this was consistent across demographic subgroups (Table 2).

**Table 1:** Prevalence of Disability Among US Adults, by Disability Measure and Selected Characteristics, National Health Interview Survey, United States, 2011–2015^a^^,^ b

Characteristic	Prevalence of Disability	
	BADQ^c^ (n = 162,551)	6Q^d^ (n = 84,757)
	Weighted %^e^^,^ f ^,^ g (95% Confidence Interval)	
**Overall**	31 .1 (30.6–31.5)	17.5 (17.1–18.0)
**Sex**		
Female	34.1 (33.6–34.7)	17.8 (17.3–18.3)
Male	27.8 (27.3–28.3)	17.2 (16.7–17.8)
**Age group, y**		
18–44	16.7 (16.3–17.1)	7.6 (7.2–8.0)
45–64	36.5 (35.8–37.1)	19.6 (18.9–20.3)
≥65	59.6 (58.8–60.4)	39.4 (38.4–40.3)
**Race/ethnicity**		
White, non-Hispanic	33.5 (32.9–34.0)	18.9 (18.3–19.5)
Black, non-Hispanic	32.0 (31.0–32.9)	19.3 (18.4–20.3)
Hispanic	23.7 (22.9–24.5)	13.0 (12.2–13.7)
Other, non-Hispanic^h^	23.0 (22.0–24.1)	12.1 (11.0–13.2)
**Education level**		
Less than high school graduate	43.6 (42.5–44.6)	30.8 (29.7–32.1)
High school graduate	36.1 (35.3–36.9)	21.0 (20.3–21.8)
Some college	30.7 (30.1–31.3)	16.2 (15.6–16.8)
College degree	21.4 (20.9–22.0)	9.6 (9.1–10.1)

Abbreviations: 6Q, 6 standard questions developed by the American Community Survey; BADQ, Basic Actions Difficulty questions.

a Among 172,465 respondents answering BADQ questions, data from respondents (n = 9,914) were excluded because of missing data on physical activity, disability status, or education level. The analytic sample included 162,551 adults.

b Among 86,276 respondents answering 6Q questions, data from respondents (n = 1,519) were excluded because of missing data on physical activity, disability status, or education level. The analytic sample included 84,757 adults.

c BADQ asked, “By yourself, and without using any special equipment, how difficult is it for you to: Walk a quarter of a mile — about 3 city blocks? Walk up 10 steps without resting? Stand or be on your feet for about 2 hours? Sit for about 2 hours? Stoop, bend, or kneel? Reach up over your head? Use your fingers to grasp or handle small objects? Lift or carry something as heavy as 10 pounds such as a full bag of groceries?” (movement difficulty); “During the past 30 days, how often did you feel: So sad that nothing could cheer you up? Nervous? Restless or fidgety? Hopeless? That everything was an effort? Worthless?” (emotional difficulty); “Do you have trouble seeing, even when wearing glasses or contact lenses?” (seeing difficulty) “Without the use of hearing aids or other listening devices, is your hearing excellent, good, a little trouble hearing, moderate trouble, a lot of trouble, or are you deaf?” (hearing difficulty); and “Are you limited in any way because of difficulty remembering or because you experience periods of confusion?” (cognitive difficulty).

d 6Q asked, “Are you deaf or do you have serious difficulty hearing” (hearing); “Are you blind, or do you have serious difficulty seeing, even when wearing glasses?” (vision); “Because of a physical, mental, or emotional condition, do you have serious difficulty concentrating, remembering, or making decisions?” (cognition); “Do you have serious difficulty walking or climbing stairs?” (mobility); “Do you have difficulty dressing or bathing?” (self-care); and “Because of a physical, mental, or emotional condition, do you have difficulty doing errands alone such as visiting a doctor's office or shopping?” (independent living).

e Sample weights reflect the probability of selection and adjustments for nonresponse and poststratification.

f All pairwise comparisons for disability prevalence between the strata of demographic characteristics in those with a disability were significantly different except between female and male sex, non-Hispanic white and non-Hispanic black races/ethnicities, and Hispanic and other races/ethnicities for BADQ and between Hispanic and other races/ethnicities for 6Q.

g Significant linear and quadratic trends in disability prevalence were observed for both measures by age and education through orthogonal comparisons (*P* < .001, except BADQ quadratic trend for education, *P* = .008).

h Other race/ethnicity includes American Indian, Alaskan Native, Asian, and multiple races.

**Table 2:** Prevalence of Inactivity Among US Adults, by Disability Measure and Selected Characteristics, National Health Interview Survey, United States, 2011–2015

Characteristic	Prevalence of Inactivity					
	BADQ^a^			6Q^b^		
	Disability (n = 55,694)	No Disability (n = 106,857)	Absolute Difference	Disability (n = 17,333)	No Disability (n = 67,424)	Absolute Difference
	Weighted %^c^^,^ d ^,^ e (95% CI)			Weighted %^c^^,^ d ^,^ e (95% CI)		
**Overall**	42.9 (42.0–43.7)	24.3 (23.7–24.9)	18.6	52.5 (51.2–53.7)	26.2 (25.6–26.9)	26.3
**Sex**						
Female	44.4 (43.5–45.4)	25.1 (24.4–25.8)	19.3	55.7 (54.1–57.3)	27.5 (26.8–28.3)	28.2
Male	40.9 (39.8–42.0)	23.5 (22.8–24.3)	17.4	48.9 (47.3–50.5)	24.8 (24.0–25.6)	24.1
**Age group, y**						
18–44	32.6 (31.3–33.9)	23.0 (22.4–23.7)	9.6	39.3 (36.9–41.7)	23.7 (22.9–24.4)	15.6
45–64	43.1 (41.9–44.3)	25.4 (24.6–26.2)	17.7	53.7 (51.9–55.5)	27.4 (26.5–28.4)	26.3
≥65	50.5 (49.4–51.6)	28.0 (26.9–29.2)	22.5	57.9 (56.2–59.6)	33.1 (31.9–34.4)	24.8
**Race/ethnicity**						
White, non-Hispanic	41.3 (40.3–42.3)	20.3 (19.6–21.1)	21.0	50.8 (49.3–52.4)	22.8 (22.0–23.6)	28.0
Black, non-Hispanic	50.2 (48.4–51.9)	31.8 (30.7–33.0)	18.4	59.3 (56.8–61.8)	33.9 (32.5–35.4)	25.4
Hispanic	47.5 (45.8–49.2)	34.6 (33.4–35.7)	12.9	57.6 (55.0–60.3)	35.2 (33.9–36.6)	22.4
Other, non-Hispanic^f^	37.8 (35.3–40.2)	24.1 (22.6–25.6)	13.7	46.6 (42.2–51.0)	25.3 (23.5–27.1)	21.3
**Education level**						
Less than high school graduate	59.1 (57.7–60.6)	43.1 (41.7–44.4)	16.0	63.5 (61.6–65.4)	44.1 (42.7–45.6)	19.4
High school graduate	49.8 (48.5–51.0)	33.8 (32.8–34.8)	16.0	57.4 (55.3–59.5)	35.7 (34.6–36.9)	21.7
Some college	37.7 (36.5–38.9)	22.4 (21.5–23.2)	15.3	47.6 (45.7–49.5)	24.0 (23.1–25.0)	23.6
College degree	25.8 (24.7–26.9)	13.4 (12.9–13.9)	12.4	34.6 (32.1–37.1)	14.4 (13.8–15.1)	20.2

Abbreviations: 6Q, 6 standard questions developed by the American Community Survey; BADQ, Basic Actions Difficulty questions; CI, confidence interval.

a BADQ asked, “By yourself, and without using any special equipment, how difficult is it for you to: Walk a quarter of a mile — about 3 city blocks? Walk up 10 steps without resting? Stand or be on your feet for about 2 hours? Sit for about 2 hours? Stoop, bend, or kneel? Reach up over your head? Use your fingers to grasp or handle small objects? Lift or carry something as heavy as 10 pounds such as a full bag of groceries?” (movement difficulty); “During the past 30 days, how often did you feel: So sad that nothing could cheer you up? Nervous? Restless or fidgety? Hopeless? That everything was an effort? Worthless?” (emotional difficulty); “Do you have trouble seeing, even when wearing glasses or contact lenses?” (seeing difficulty) “Without the use of hearing aids or other listening devices, is your hearing excellent, good, a little trouble hearing, moderate trouble, a lot of trouble, or are you deaf?” (hearing difficulty); and “Are you limited in any way because of difficulty remembering or because you experience periods of confusion?” (cognitive difficulty).

b 6Q asked, “Are you deaf or do you have serious difficulty hearing” (hearing); “Are you blind, or do you have serious difficulty seeing, even when wearing glasses?” (vision); “Because of a physical, mental, or emotional condition, do you have serious difficulty concentrating, remembering, or making decisions?” (cognition); “Do you have serious difficulty walking or climbing stairs?” (mobility); “Do you have difficulty dressing or bathing?” (self-care); and “Because of a physical, mental, or emotional condition, do you have difficulty doing errands alone such as visiting a doctor's office or shopping?” (independent living).

c Sample weights reflect the probability of selection and adjustments for nonresponse and poststratification.

d Inactivity prevalence was significantly different (*P* < .05) between the strata of demographic characteristics in those with and without a disability except between non-Hispanic black and Hispanic races/ethnicities in those both with and without disability for 6Q. Demographic inactivity comparisons for each strata between disability and no disability were significantly different (*P* < .001).

e Regardless of measure used, significant linear and quadratic trends in inactivity prevalence were observed by age and education among those with disability through orthogonal comparisons (*P* < .001, except BADQ quadratic trend for education, *P* = .02).

f Other race/ethnicity includes American Indian, Alaskan Native, Asian, and multiple races.

Patterns in inactivity prevalence by sex, age, race/ethnicity, and education were similar by disability measure (Table 2). Inactivity prevalence was higher for female respondents than male respondents and increased with age. By race/ethnicity, inactivity was highest among non-Hispanic blacks (BADQ, 50.2%; 6Q, 59.3%) followed by Hispanics (BADQ, 47.5%; 6Q, 57.6%), non-Hispanic whites (BADQ, 41.3%; 6Q, 50.8%), and other races/ethnicities (BADQ, 37.8%; 6Q, 46.6%). Inactivity prevalence decreased as education level increased.

## Discussion

Prevalence of inactivity among adults with disabilities is higher than among those without disabilities, and we found that the magnitude of inactivity prevalence differed by disability measure. Inactivity estimates were higher among those with disability measured by 6Q (53%) than among those measured by BADQ (43%). Understanding how different measures can influence prevalence estimates is important for public health planning.

Estimates of disability prevalence in US adults have ranged from 20% to 30% (7–9). Our estimates from BADQ and 6Q are in the high and low end of this range, respectively. Although 6Q and BADQ cover similar domains of disability, they differ in defining severity. Many questions from BADQ use Likert-type responses, and individuals are categorized as having a disability when they report at least some difficulty with one or more activities in a domain; most domains in 6Q use “serious” as a qualifier. Therefore, individuals with less severe disabilities may be identified with the BADQ measure, resulting in a higher prevalence estimate (7).

A paradox between the measures was observed; analyses of surveillance data should consider the potential influence of choice of measure on the magnitude of health indicators among people with disability. Higher inactivity prevalence was noted when 6Q was used, which may be explained by differences in disability severity captured by each measure. Individuals with more severe disabilities tend to be less active (10,11); therefore, measures that ascertain more severe disability are likely to result in higher inactivity prevalence. Although inactivity prevalence varied by disability measure, demographic patterns were consistent across measures. Furthermore, regardless of the disability measure used, at least 40% of respondents with disabilities were inactive.

These results are subject to at least 3 limitations. First, NHIS data are self-reported and may be subject to recall and social desirability biases. Second, individual perception of disability may be inconsistent between respondents; therefore, an individual may be considered to have a disability by one measure and not another. Furthermore, for some sampled adults, questions are answered by a proxy who may have different perceptions of the individual’s disability. Finally, 6Q was asked of only half of the sample adults. However, both samples were weighted to be nationally representative, and analyses limited to those with information on both sets of questions yielded disability estimates similar to the full sample.

Disability is measured in various ways, and differences in prevalence can influence estimates of health-related indicators in adults with disability. Considering the disability definition used is important when drawing conclusions and allocating resources for public health programs. Both measures indicated a large prevalence of physical inactivity, and these findings reinforce the necessity of including adults with disabilities in physical activity promotion activities (12).
